# Neutrophil Depletion Attenuates Placental Ischemia-Induced Hypertension in the Rat

**DOI:** 10.1371/journal.pone.0132063

**Published:** 2015-07-02

**Authors:** Jean F. Regal, Kathryn E. Lillegard, Ashley J. Bauer, Barbara J. Elmquist, Alex C. Loeks-Johnson, Jeffrey S. Gilbert

**Affiliations:** Department of Biomedical Sciences, University of Minnesota Medical School Duluth, Duluth, Minnesota, United States of America; Center for Human Reproduction, UNITED STATES

## Abstract

Preeclampsia is characterized by reduced placental perfusion with placental ischemia and hypertension during pregnancy. Preeclamptic women also exhibit a heightened inflammatory state and greater number of neutrophils in the vasculature compared to normal pregnancy. Since neutrophils are associated with tissue injury and inflammation, we hypothesized that neutrophils are critical to placental ischemia-induced hypertension and fetal demise. Using the reduced uteroplacental perfusion pressure (RUPP) model of placental ischemia-induced hypertension in the rat, we determined the effect of neutrophil depletion on blood pressure and fetal resorptions. Neutrophils were depleted with repeated injections of polyclonal rabbit anti-rat polymorphonuclear leukocyte (PMN) antibody (antiPMN). Rats received either antiPMN or normal rabbit serum (Control) on 13.5, 15.5, 17.5, and 18.5 days post conception (dpc). On 14.5 dpc, rats underwent either Sham surgery or clip placement on ovarian arteries and abdominal aorta to reduce uterine perfusion pressure (RUPP). On 18.5 dpc, carotid arterial catheters were placed and mean arterial pressure (MAP) was measured on 19.5 dpc. Neutrophil-depleted rats had reduced circulating neutrophils from 14.5 to 19.5 dpc compared to Control, as well as decreased neutrophils in lung and placenta on 19.5 dpc. MAP increased in RUPP Control vs Sham Control rats, and neutrophil depletion attenuated this increase in MAP in RUPP rats without any effect on Sham rats. The RUPP-induced increase in fetal resorptions and complement activation product C3a were not affected by neutrophil depletion. Thus, these data are the first to indicate that neutrophils play an important role in RUPP hypertension and that cells of the innate immune system may significantly contribute to pregnancy-induced hypertension.

## Introduction

Preeclampsia and related hypertensive disorders of pregnancy affect up to 10% of pregnancies in the United States and are a leading cause of maternal death and medically indicated preterm birth. Clinical presentation of preeclampsia is characterized by new onset hypertension after 20 weeks of pregnancy, often with proteinuria. Preeclampsia can result in multiorgan damage and inflammation that can significantly impact the health of both mother and child [1]. Clinical diagnosis of preeclampsia is often difficult due to the lack of specific biomarkers, and the only definitive treatment for preeclampsia is delivery of the placenta. Antihypertensive therapy is employed to minimize complications for the mother and to prolong the pregnancy to avoid preterm birth. The cause of preeclampsia is unknown, but impaired spiral artery remodeling is observed in the placenta resulting in placental ischemia. This reduced placental perfusion is associated with myriad changes [2–4] including imbalance between pro-angiogenic (e.g. vascular endothelial growth factor, VEGF) and anti-angiogenic (e.g. soluble fms-like tyrosine kinase-1, sFlt-1; soluble endoglin, sEng) factors in the maternal circulation [5], endothelial dysfunction, and endothelin pathway activation.

The immune system is increasingly recognized as important in the pathophysiology of preeclampsia. Changes in the adaptive immune response occur in normal pregnancy to sustain the pregnancy yet still afford some protection from infection. These changes include decreased Th17 cells and increased Tregs and a shift to favor Th2 immunity over Th1 immunity compared to a healthy non-pregnant female [6, 7]. Such changes are thought to help ensure a successful pregnancy but potentially increase susceptibility to intracellular infections during pregnancy that are dependent on Th1 T cell for defense [7]. In preeclampsia, a decrease in the Treg/Th17 ratio occurs with fewer Treg and/or more Th17 cells compared to a normal pregnancy [8] with accumulating evidence suggesting that these changes contribute to pregnancy complications. The innate immune response is also affected during normal pregnancy compared to non-pregnant women, with a heightened inflammatory state and increased complement activation [9] that is accentuated even more in preeclampsia [10]. In addition, as pregnancy progresses from first to third trimester, increased numbers of neutrophils are noted in the maternal circulation [11, 12], with elevated neutrophil counts in preeclampsia compared to uncomplicated pregnancies at term. Evidence also suggests neutrophils in preeclampsia are activated with increased concentrations of the neutrophil granule product elastase released into the maternal circulation and detectable in the placenta compared to a normal pregnancy [13, 14]. Though a limited degree of neutrophil activation is part of a normal pregnancy [15], excessive inflammation, neutrophil activation and oxidative stress may contribute to numerous adverse pregnancy outcomes [16–22]. Neutrophil infiltration in the systemic vasculature, as measured by the percentage of blood vessels of subcutaneous fat staining for CD66b, increased in normal pregnancy over non-pregnant women with an even greater increase in preeclampsia [23]. In addition, neutrophil activation as measured by intracellular reactive oxygen species or myeloperoxidase was heightened in pregnant over that of non-pregnant with again a greater increase in preeclampsia compared to normal pregnant [15, 23, 24]. In animal models, the first description of the importance of the neutrophil in fetal demise was in the CBAxDBA/2 model of recurrent spontaneous abortion [25]. Subsequently, studies in a mouse model of anti-phospholipid syndrome and spontaneous miscarriage also found an important role for neutrophils in fetal demise [26]. However, neutrophils are not required for preterm birth in infection-induced preterm labor [27] with data trends even suggesting that neutrophils may protect against preterm birth. Rinaldi and colleagues [27] used LPS-induced preterm birth in the mouse as a model of infection induced preterm delivery, hypothesizing that depleting neutrophils would prevent LPS-induced preterm delivery. Surprisingly, neutrophil depletion did not prevent LPS induced preterm birth, but actually resulted in a nonsignificant trend toward even earlier delivery. It is possible that neutrophil depletion could result in LPS recruitment of a greater proportion of activated neutrophils to the decidua leading to earlier delivery.

Hypertension has recently been associated with inflammation and oxidative stress as well as involvement of the immune system with many studies focusing on the lymphocyte [28, 29]. However, evidence also suggests that neutrophils may contribute to hypertension. In the Sabra rat salt sensitive model of hypertension, depletion of neutrophils significantly attenuates hypertension [30]. Whether neutrophils contribute to the hypertension and fetal demise resulting from placental ischemia in pregnancy has not been tested. Thus, we hypothesized that neutrophils are a significant contributor to the development of hypertension and fetal resorptions following placental ischemia. To test this hypothesis we used the reduced utero-placental perfusion pressure (RUPP) model of placental ischemia-induced hypertension in the pregnant rat. Reduction of blood flow to the placenta by a surgical technique at the beginning of the third trimester results in hypertension in the mother, increased fetal resorptions and complement activation, and angiogenic imbalance as indicated by a consistent decrease in circulating VEGF with restoration of the normal blood pressure by infusion with recombinant VEGF [31–33]. Our studies were designed to determine if reduction of circulating neutrophils in the third trimester by treatment with antisera to rat neutrophils would attenuate development of hypertension.

## Materials and Methods

### Reduced utero-placental perfusion pressure (RUPP) procedure

Placental ischemia was achieved using the reduced utero-placental perfusion pressure (RUPP) procedure in the third trimester pregnant rat as previously described [31, 32]. Timed pregnant Sprague Dawley dams (Crl:CD IGS, Charles River Laboratories, Portage, MI) were anesthetized with isoflurane on 14.5 days post conception (dpc) with the date of vaginal plug designated as 0.5 dpc. A ventral midline incision was made, the lower abdominal aorta isolated and a sterile silver clip (0.203 mm ID) placed around the aorta above the iliac bifurcation. Both right and left uterine arcades were also clipped at the ovarian end, directly before the first segmental artery, using a silver clip (0.100 mm ID) to prevent compensatory blood flow to the placenta. Uterine perfusion pressure is reduced by approximately 40% using this procedure [34]. For the comparison group, a Sham operation differing only in the absence of the clips was also conducted.

### Ethics statement

All animal protocols were approved by the University of Minnesota Institutional Animal Care and Use Committee (University Animal Assurance No. A3456-01; AAALAC Accreditation December 19, 2012; University of Minnesota Animal Protocol #1104A98162) in accordance with the recommendations in the Guide for the Care and Use of Laboratory Animals of the National Institutes of Health guidelines. All surgical procedures were conducted under isoflurane anesthesia and all measures taken to ameliorate suffering.

### Experimental design

**Fig 1:**
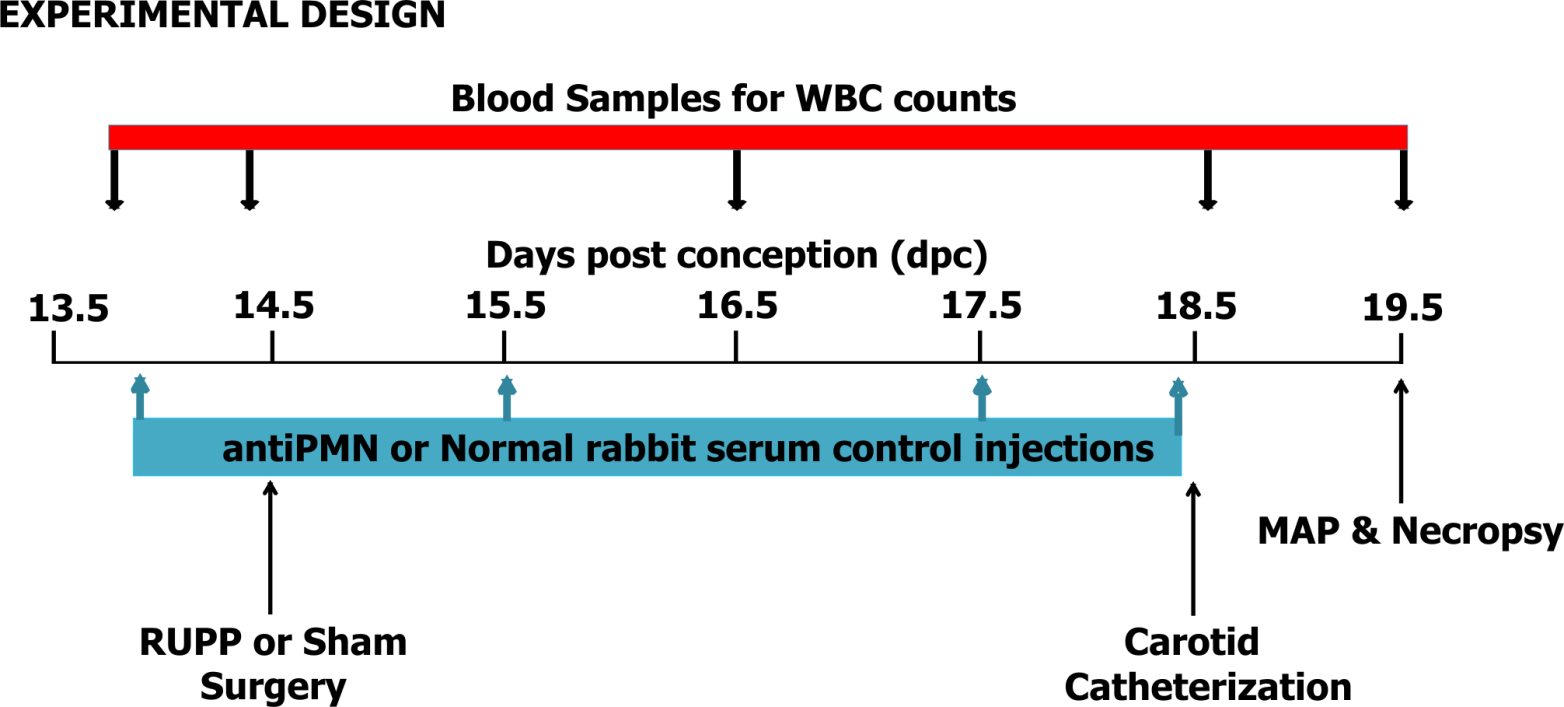
Experimental Design. Timing of injections, blood sampling and surgeries with days post conception (dpc) in timed pregnant Sprague Dawley rats. Necropsy and measurement of mean arterial pressure (MAP) occurred on 19.5 dpc.

The experimental design is depicted in Fig 1. On 13.5 dpc, 12–18 hr prior to RUPP or Sham surgery, rats were bled by saphenous vein for white cell and platelet counts. Rats were then administered an ip injection of either normal rabbit serum as Control or rabbit polyclonal antibody to rat neutrophils that had been adsorbed with red blood cells (antiPMN; AIAD51140; Accurate Chemical Co, Westbury NY). The initial dose at 13.5 dpc was given at 0.1 ml/100g body weight. Additional ip injections of Control or antiPMN were administered at 0.15ml/100g body weight on 15.5, 17.5, and 18.5 dpc. Sequential saphenous vein bleeds were used to assess the effectiveness and specificity of the antisera in depleting neutrophils and not other circulating cell populations over the third trimester. Rats were randomly assigned to one of four experimental groups based on surgical procedure and antibody treatment: 1) RUPP surgery receiving normal rabbit serum (RUPP Control, n = 14); 2) RUPP surgery receiving antiPMN (RUPP antiPMN, n = 9); 3) sham surgery receiving normal rabbit serum (Sham Control, n = 6); 4) sham surgery receiving antiPMN (Sham antiPMN, n = 6). The n for each outcome measured is indicated in each of the fig legends, dependent on samples available from each animal.

### Measurement of mean arterial pressure and tissue collection

On 18.5 dpc, a carotid artery cannula was aseptically placed under isoflurane anesthesia for measurement of mean arterial pressure (MAP) on 19.5 dpc in an unanesthetized restrained rat [31, 32]. Heparin was not used in cannulas due to its ability to inhibit complement system activation, and increase myeloperoxidase and antiangiogenic factor release into circulation [35, 36]. A 25% dextrose lock solution in sterile pyrogen free saline was used to maintain cannula patency. Following measurement of blood pressure, serum, plasma and tissues were collected in necropsy as described [33, 34, 37, 38]. The uterus was exteriorized, the total number of viable and resorbed pups counted and the pups and placentae of the right horn weighed. From the left horn, select utero-placental units were fixed in 10% neutral buffered formalin for histological analysis and assessment of neutrophil depletion.

### Assessment of neutrophil depletion

Blood was collected in EDTA coated tubes and total white blood cells and platelets counted by standard methods in a hemacytometer. Hematocrit was determined on 18.5 and 19.5 dpc with blood obtained from the carotid and aorta, respectively. Differential counts were obtained from blood smears stained with a modified Wrights’ stain (Diff Quik, American Scientific Products, McGraw Park, IL). Two hundred cells were counted and identified as eosinophils, neutrophils, lymphocytes or monocytes. Monocytes and eosinophils typically comprised less than 1% of the cell differential in all of the treatment groups so were not included in the analysis. For estimates of the numbers of neutrophils in tissue, the left lung lobe was homogenized and processed as previously described for determination of myeloperoxidase (MPO) activity [39]. MPO activity was expressed as Units MPO/mg protein in the tissue homogenate using the BioRad DC protein assay (BioRad, Hercules, CA) for protein determinations following detergent solubilization of MPO. To determine effectiveness of neutrophil depletion in the placenta, immunohistochemistry was done on 4μ sections from formalin fixed tissues. For immunohistochemical staining, placental sections were deparaffinized and rehydrated, antigen retrieval accomplished with 95 degree water bath using Rodent Decloaker (Biocare Medical RD913L, Concord, CA) for 40 minutes. Endogenous peroxides were blocked at room temperature with Envision Flex Peroxidase Blocking Reagent (Dako 2348ZCG, Carpenteria, CA). After non-specific proteins were blocked using Background Sniper (Biocare Medical BS966H) slides were incubated at 4 degrees overnight in primary antibody CD43 clone W3/13 (Biolegend 20280, SanDiego, CA) diluted 1/100 in wash buffer. Secondary antibody/detection Mouse-on-Rat HRP Polymer (Biocare Medical MRT621G) was applied and slides were incubated for 30 minutes at room temperature, followed by wash and DAB chromogen (Dako2355KRB) for 5 minutes. Slides were counterstained with hematoxylin. For assessment of granulocytic CD43 positive cells, eight areas each of placental labyrinth, junctional zone and decidua from each animal were counted and averaged from randomly generated images. Since CD43 may also label monocytes and lymphocytes, granulocytes were also distinguished morphologically from mononuclear cells in the placenta.

### Complement measurements

Serum concentrations of C3a were measured by Western immunoblot as previously described [31, 40]. The primary antibody used for immunodetection was IgG fraction of rabbit polyclonal antibody to the 9 carboxy-terminal amino acids of rat C3a (Research Genetics, Inc., Huntsville, AL). The blot was probed with a 1:2,500 dilution of primary antibody followed by 80 ng/mL of goat anti-rabbit IgG coupled to horseradish peroxidase (Pierce, Rockford, IL). After incubation with SuperSignal West Femto Maximum Sensitivity Substrate (Thermo Fisher Scientific, Rockford, IL) for 5 minutes, images were captured with FluoroChem camera (AlphaInnotech, San Leadro, CA), and pixel density quantification with Image J (public domain program developed at NIH). Dilutions of a standard pool of rat serum complement activated by yeast were used to construct a standard curve on each gel and a regression equation was used to calculate the relative amount of C3a in the unknown samples. Relative amounts of C3a in each sample were expressed as C3a units/μl based on signal intensity of 1 μl of standard pool of rat serum activated by yeast. Rat C5a was not detectable in yeast-activated serum by current assay methods. The inverse dilution of serum that causes 50% hemolysis of sensitized sheep erythrocytes (CH_50_) was also determined as an indicator of total complement pathway function as previously described [31].

### Plasma VEGF and oxidative stress

Circulating free VEGF concentrations in EDTA plasma collected on 19.5 dpc were measured using commercially available kit for Mouse VEGF from R&D Systems (Quantikine, Minneapolis, MN) according to manufacturer’s directions and as described previously [31]. A Trolox- equivalent antioxidant capacity of the plasma was determined with a total antioxidant assay (Cayman Chemical, Ann Arbor, MI). Thiobarbituric acid reactive substances assay (Cayman Chemical, Ann Arbor, MI) was performed to assess oxidative stress in plasma, placenta and kidney with an end measurement of malondialdehyde (MDA) according to the manufacturer’s instructions as previously described [41].

### Statistical analyses

Data are presented as mean or geometric mean ± SE, and differences were considered significant when p<0.05. Data were analyzed using two-way ANOVA with three individual contrasts considered most relevant for comparison of means: Sham Control vs RUPP Control, RUPP Control vs RUPP antiPMN, and Sham Control vs Sham antiPMN. C3a, CH_50_ and VEGF values were logged to meet model assumptions. For analysis of changes in % neutrophils over the 13.5 to 19.5 dpc, repeated measures analysis of variance was used since sequential blood samples from each animal cannot be considered independent measures. All statistical analyses used JMP or SAS software (SAS Institute, Cary, NC).

## Results

### Neutrophil depletion attenuates placental ischemia-induced hypertension

**Fig 2:**
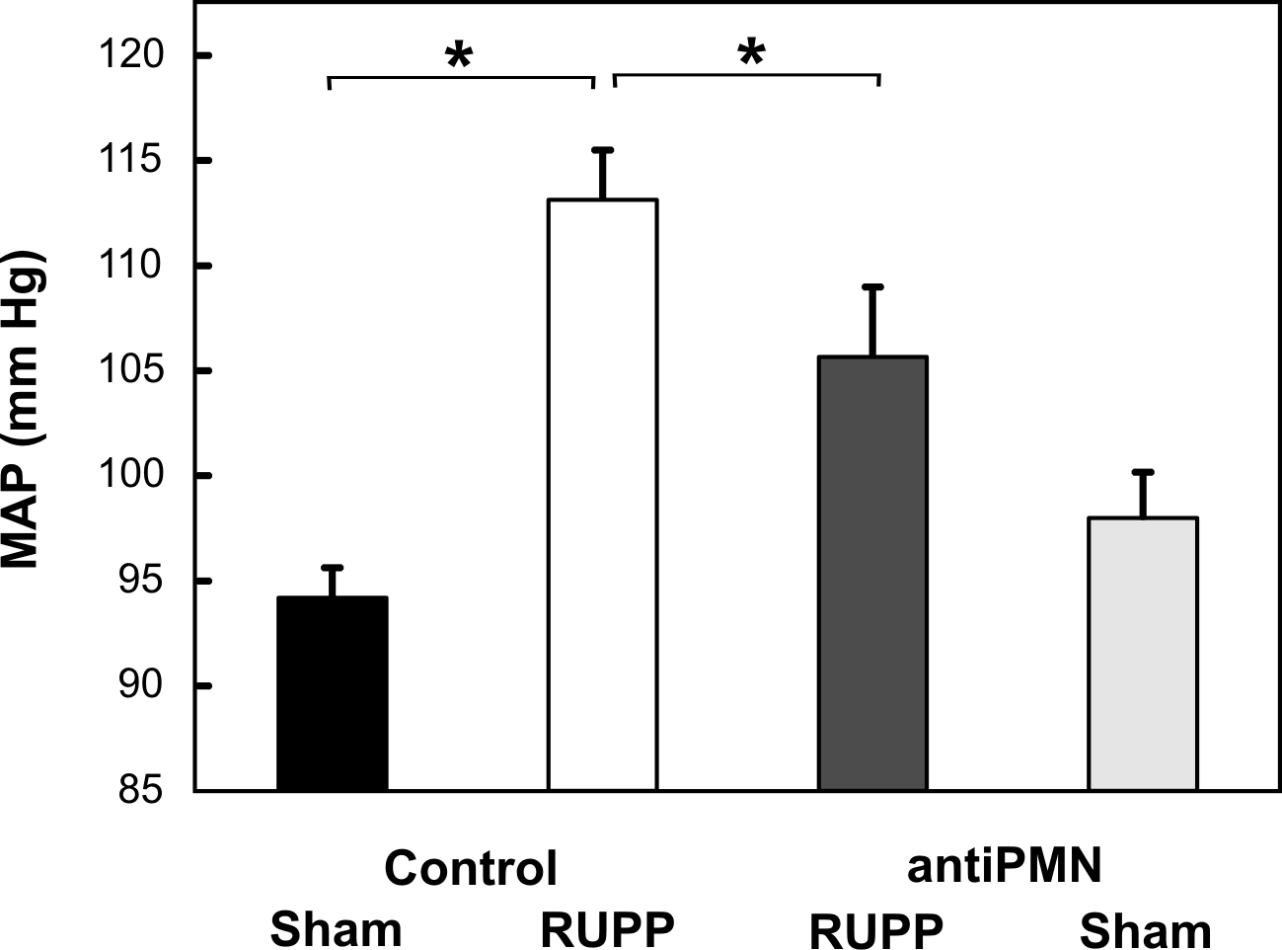
Neutrophil depletion significantly attenuates placental ischemia-induced increase in mean arterial pressure (MAP). Animals were treated with normal rabbit serum (Control) or antiPMN antibody from 13.5–18.5 dpc. The increase in MAP in RUPP Control (n = 14) compared to Sham Control (n = 5) was significantly inhibited by neutrophil depletion (RUPP antiPMN, n = 9). MAP did not differ between Sham antiPMN (n = 5) and Sham Control groups. Values represent mean ± SE of MAP measured 19.5 dpc. *p<0.05 for indicated comparison.

**Fig 3:**
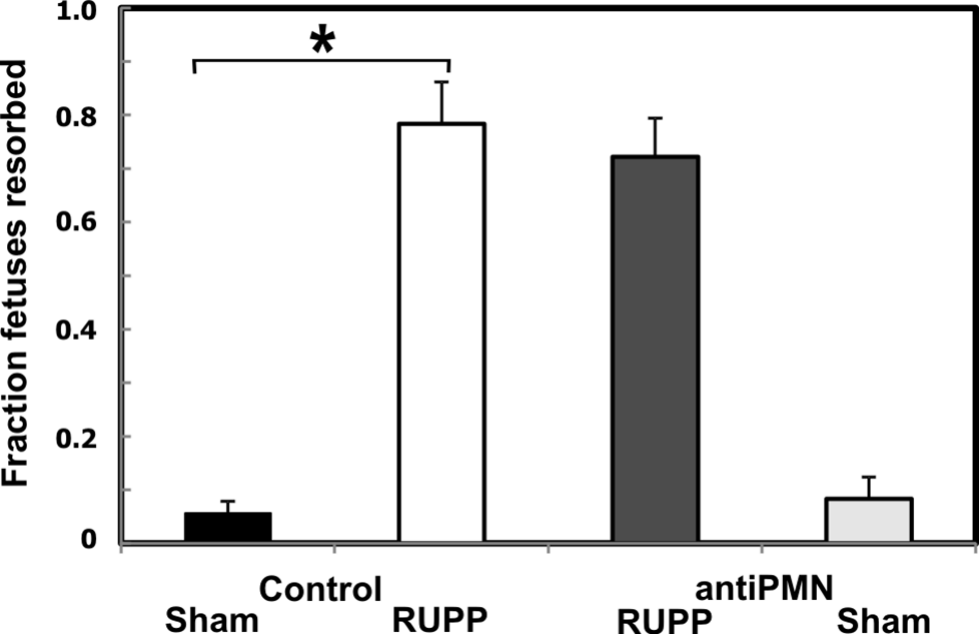
Neutrophil depletion does not affect placental ischemia-induced fetal resorptions. Animals were treated with normal rabbit serum (Control) or antiPMN antibody from 13.5–18.5 dpc. The increase in fetal resorptions in RUPP Control (n = 14) compared to Sham Control (n = 6) was not affected by neutrophil depletion (RUPP antiPMN, n = 9). Fetal resorptions did not differ between Sham antiPMN (n = 6) and Sham Control groups. Values represent mean ± SE of the fraction of resorbed fetuses on 19.5 dpc of gestation. *p<0.05 for indicated comparison.

Placental ischemia (RUPP) resulted in a clear increase in mean arterial pressure at 19.5 dpc (Fig 2). Treatment with antiPMN antibody significantly attenuated this blood pressure increase but did not significantly alter blood pressure in Sham animals. Fetal resorptions following placental ischemia were significantly higher than sham as expected (Fig 3) but antiPMN treatment did not significantly improve fetal survival. Average fetal weight was decreased with RUPP surgery (2.20±0.09 g, Sham; 2.09±0.10 g, RUPP) but did not reach statistical significance in this cohort and was not affected by antiPMN treatment (2.23±0.07, Sham antiPMN; 2.11±0.06, RUPP antiPMN). Average placental weight was also not altered by RUPP in this cohort (0.41±0.03 g, Sham; 0.43±0.02 g, RUPP) with no affect of antiPMN treatment (0.43±0.01 g, Sham antiPMN; 0.38±0.01 g, RUPP antiPMN).

### Neutrophil depletion does not affect complement activation or decreased VEGF following placental ischemia

**Fig 4:**
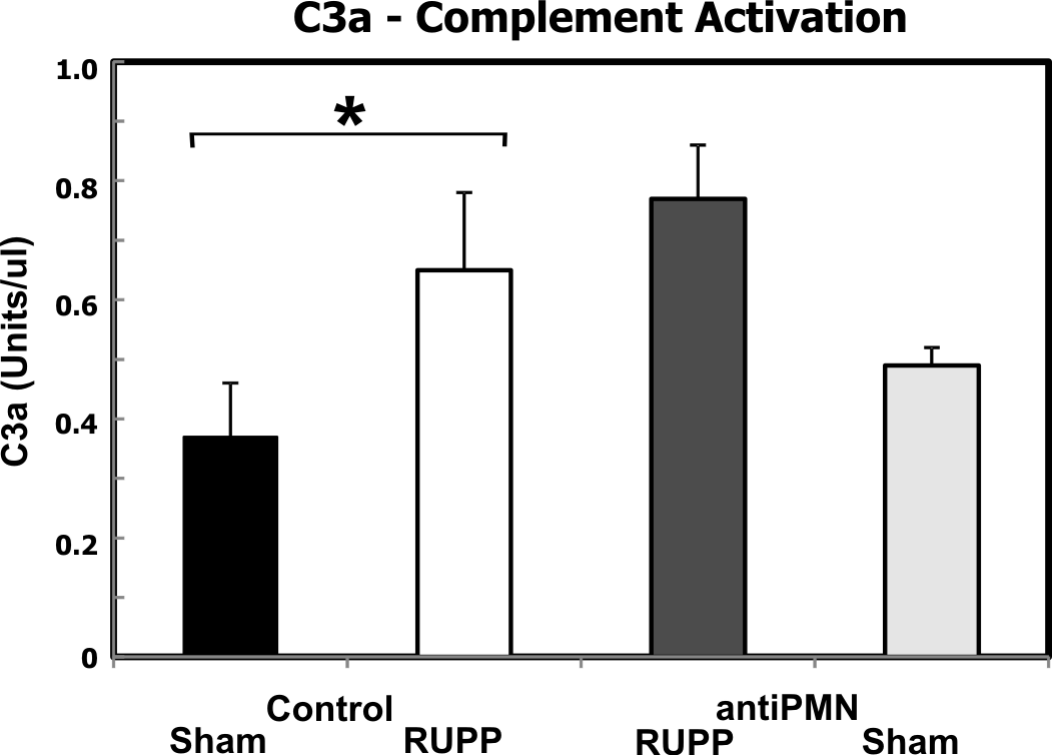
Neutrophil depletion does not affect placental ischemia-induced increase in C3a. Animals were treated with normal rabbit serum (Control) or antiPMN antibody from 13.5–18.5 dpc. *p<0.05 for indicated comparison. The increase in serum C3a in RUPP Control (n = 10) compared to Sham Control (n = 4) was not affected by neutrophil depletion (RUPP antiPMN, n = 7). C3a did not significantly differ between Sham antiPMN (n = 4) and Sham Control groups. Values represent geometric mean ± SE of C3a units/ μl in serum obtained 19.5 dpc. Units of C3a are relative to a standard pool of yeast activated rat serum as described in Methods.

Our previous studies demonstrated that complement activation, particularly activation products C3a and C5a, were critical for placental ischemia-induced hypertension [31, 32] with increased complement activation evident following placental ischemia. Administration of antiPMN antibody could result in immune complex-mediated complement activation when clearing neutrophils, leading to generation of C3a and C5a that can interact with their respective receptors on neutrophils. Thus, we assessed the effect of antiPMN treatment on complement activation as measured by C3a generation and on total hemolytic complement activity. As seen in Fig 4, placental ischemia significantly increased C3a generation as previously reported, and antiPMN treatment did not significantly affect the increase in C3a. In addition, total hemolytic complement activity in serum of the 4 treatment groups was not significantly affected by antiPMN treatment (S1 File), indicating that immune complexes were not activating and depleting complement, leading to the attenuation of blood pressure by antiPMN treatment.

Previous studies had also demonstrated a role for decreased VEGF in placental ischemia-induced hypertension [33] so the effect of neutrophil depletion on plasma VEGF was determined. These studies were done prior to publication of the study by Weissgerber [42] questioning the validity of the VEGF ELISA assay for rat VEGF. Placental ischemia significantly reduced circulating plasma VEGF as determined by the R&D ELISA kit with 834 ng/ml in RUPP Control animals (n = 13) compared to 1384 ng/ml in Sham Control (n = 6). The concentration of VEGF in RUPP antiPMN was 720 ng/ml (n = 9). This was not significantly different from RUPP Control animals. VEGF concentration in Sham antiPMN (1148 ng/ml; n = 6) was not significantly different from Sham Control. These data suggest that changes in plasma VEGF operated in parallel to neutrophils in leading to increased blood pressure following placental ischemia. Alternatively, changes in VEGF could lead to involvement of neutrophils in hypertension. Future studies assessing the validity of the VEGF ELISA in the rat are certainly warranted given the recent studies.

When activated, neutrophils are certainly a source of oxidative stress and are increased in systemic blood vessels of women with preeclampsia [43]. To assess oxidative stress, we measured MDA production in plasma, placenta and kidney as well as plasma antioxidant capacity. MDA production in plasma was not increased in RUPP vs Sham (9.7±0.5 μM MDA, RUPP vs 12.8±3.5 μM MDA, Sham) and antioxidant capacity was also not different (1.52±0.18 mM, RUPP vs 1.48±0.10 mM, Sham). Treatment with antiPMN antibody did not alter either MDA production in plasma nor the antioxidant capacity (S1 File). In addition, we assessed MDA production in both placenta and kidney and saw no differences in RUPP vs Sham, with or without antiPMN treatment (S1 File).

### Effectiveness and specificity of neutrophil depletion

**Fig 5:**
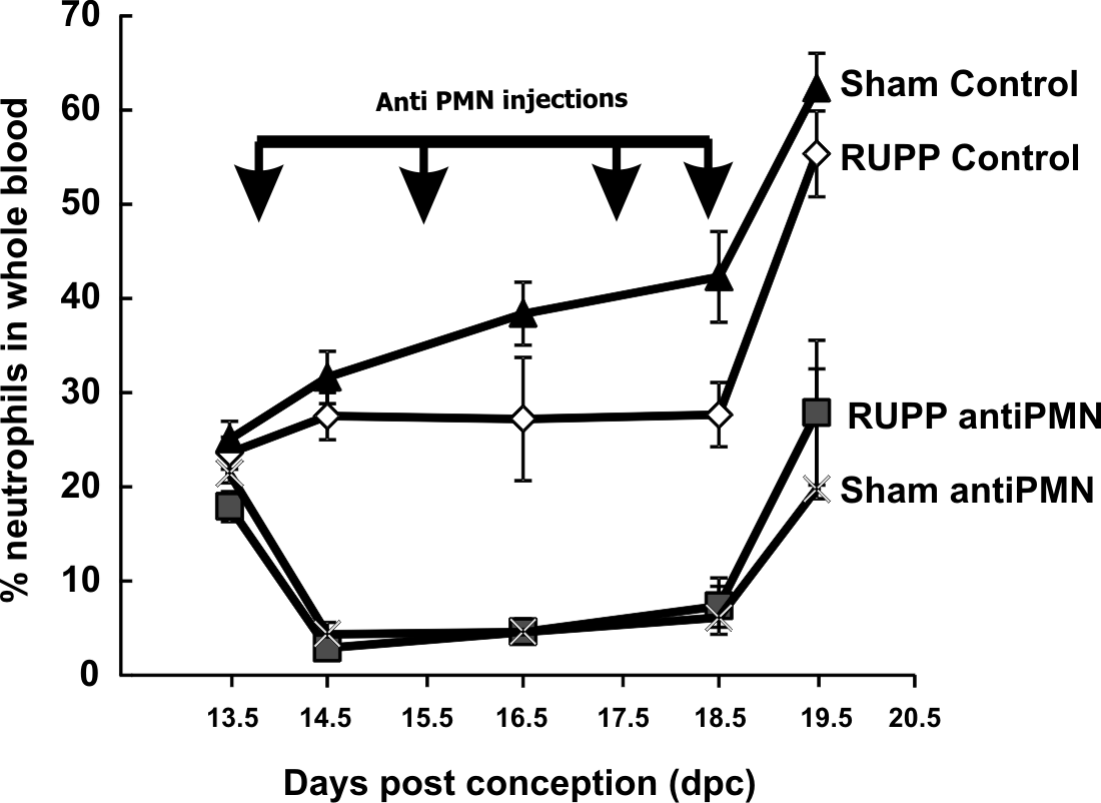
Treatment with antiPMN antibody 13.5–18.5 dpc reduces the percentage of circulating neutrophils. Animals were treated with normal rabbit serum (Control) or antiPMN antibody as depicted by arrows. AntiPMN treatment significantly reduced circulating neutrophils compared to normal rabbit serum treatment. Values represent mean ± SE of % neutrophils in blood collected from 5–14 animals. Blood samples were taken prior to first injection (13.5 dpc) and prior to RUPP or Sham surgery (14.5 dpc), at 16.5 dpc, at 18.5 dpc with carotid cannulation and at necropsy (19.5 dpc).

The effectiveness of treatment with antiPMN antibody was assessed by determining numbers of circulating neutrophils throughout the treatment period, as well as evaluating neutrophils in placenta and lung on 19.5 dpc at time of necropsy. The lung was chosen for evaluation based on data indicating that neutrophils may sequester in small vessels in the pulmonary circulation when activated. Total WBC and differential counts revealed that neutrophils were effectively decreased by antiPMN treatment in the circulation on 14.5 dpc at the time of the RUPP or Sham surgery (Fig 5). With additional antibody injections on both 15.5 dpc and 17.5 dpc, circulating neutrophils were still significantly decreased in antiPMN treated animals compared to Control. An additional antiPMN treatment was administered at 18.5 dpc prior to carotid artery cannulation in all animals under aseptic conditions. At necropsy, under isoflurane anesthesia, animals were bled via the aorta into EDTA and cell counts again determined. In all animal groups, the numbers of neutrophils increased at 19.5 dpc at time of necropsy after MAP determination. Repeated measures analysis of variance detected no significant difference comparing changes in % neutrophils in Sham Control compared to RUPP Control. In Fig 5, data were expressed as % neutrophils, but similar results were apparent when total neutrophils were assessed as well (S2 File). Limited experiments in animals that were not cannulated for carotid artery MAP measurement suggested that the increase in % neutrophils on 19.5 dpc was likely an acute response to carotid cannulation on 18.5 dpc.

Verifying the specificity of the neutrophil depletion, antiPMN treatment did not significantly change platelet numbers over the time course compared to control (S1 Fig 1). As expected, the % lymphocytes increased with antiPMN treatment (S1 Fig 2) and subsequently decreased in all treatment groups at 19.5 dpc, coincident with the acute increase in % neutrophils. Monocytes and eosinophils typically comprised less than 1% of the cell differential in all of the treatment groups so were not included in the analysis. An occasional band neutrophil was identified, but provided no evidence of immature neutrophils in the circulation with either normal rabbit serum or antiPMN treatment. Hematocrit was only assessed on 18.5 dpc and 19.5 dpc from carotid and aortic bleeds, respectively, to minimize the volume of blood taken over the course of the third trimester. No differences in hematocrit between treatment groups was detected on 18.5 dpc and 19.5 dpc indicating red blood cells were not being depleted by the treatments (S1 File).

**Fig 6:**
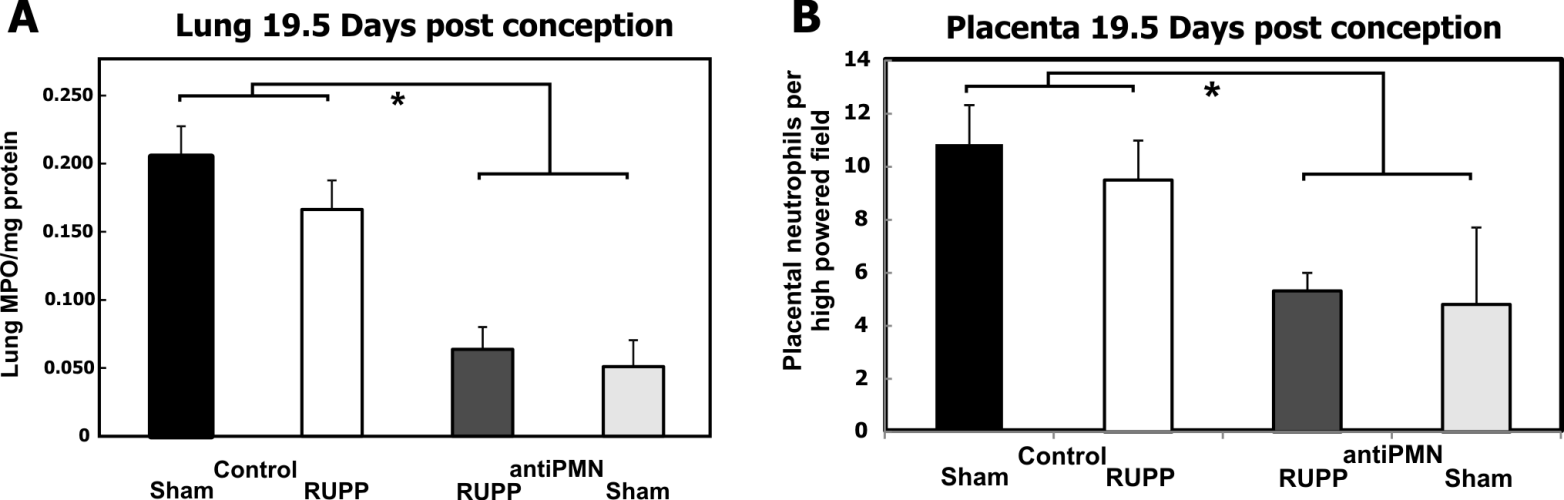
Treatment with antiPMN antibody reduces lung myeloperoxidase (MPO) activity and reduces neutrophils in placenta. Animals were treated with normal rabbit serum (Control) or antiPMN antibody from 13.5–18.5 dpc. *p<0.05 for antiPMN effect by analysis of variance. A). Lung MPO was used as an indicator of the number of neutrophils in the lung. AntiPMN treatment significantly reduced MPO compared to normal rabbit serum treatment. Values represent mean ± SE of Units of lung MPO activity per mg protein of lung homogenate in lungs collected from 5–14 animals on 19.5 dpc. B). AntiPMN treatment significantly reduced the number of neutrophils in placenta compared to normal rabbit serum treatment. Values represent mean ± SE of placental neutrophils per high-powered field in placenta collected from 3–6 animals on 19.5 dpc.

In view of the increase in circulating neutrophils on 19.5 dpc at time of necropsy, we also evaluated neutrophils in the tissue. Lungs were homogenized and MPO determined as an indicator of number of neutrophils sequestered in the lung. As seen in Fig 6A, antiPMN treatment significantly decreased the neutrophils detectable in lung at necropsy on 19.5 dpc in both RUPP and Sham groups. Determination of MPO in the placenta is complicated by numerous other interfering peroxidases and inhibitory components. Because of this, immunohistochemistry was utilized to directly assess the number of neutrophils in placenta at 19.5 dpc in the treatment groups. As seen in Fig 6B, the number of neutrophils in the placenta was decreased by antiPMN treatment at 19.5 dpc. Thus, the increase in circulating neutrophils at 19.5 dpc following carotid cannulation was not reflected in the lung and placenta.

## Discussion

The data in this paper shows for the first time that the neutrophil contributes to the hypertensive response following placental ischemia, indicating a role for a specific cellular component of the innate immune system. In addition, our observation that neutrophil depletion did not alter blood pressure in the sham group indicates that the neutrophil does not play a role in the blood pressure changes occurring in a normal pregnancy. Lastly, neutrophil depletion did not modify the RUPP-induced change in VEGF or fetal growth and viability indicating a different mechanism leads to these outcomes compared to the pathophysiology leading to hypertension.

Our studies clearly demonstrate that neutrophils contribute to hypertension following placental ischemia, with neutrophil depletion significantly attenuating the increase in mean arterial pressure following placental ischemia in the rat. Previous studies of LaMarca et al have demonstrated that depletion of B cells with high doses of rituximab attenuates the placental ischemia-induced hypertension in the rat [44]. Her continued studies suggest that CD4+ T cells are also in part responsible for the hypertension following placental ischemia since adoptive transfer of CD4+ T cells from the RUPP rat results in hypertension in a normal pregnant rat [45]. Subsequently, Wallace et al [46] have demonstrated a role for CD4+ T cells in the generation of oxidative stress in the kidney and placenta in mediating placental ischemia-induced hypertension as well. Thus, cell types associated with both the adaptive and innate immune system contribute to placental ischemia-induced hypertension.

Neutrophils are normally increased in pregnancy as well as in preeclampsia [47]. However, in the placenta and circulation of preeclamptic patients, the neutrophil products myeloperoxidase and elastase are increased compared to normal pregnancy [13, 35] suggesting a heightened state of activation of neutrophils. In addition, neutrophils, but not lymphocytes or monocytes, infiltrate blood vessels of subcutaneous fat in women with preeclampsia [43] suggesting they may contribute to the vascular and endothelial dysfunction in preeclampsia. Shed placental microparticles have been shown to activate neutrophils, promoting formation of neutrophil extracellular traps or NETs [48] that are present in the intervillous space in preeclamptic placentas. Recent studies in normal human placenta also indicate that normal density granulocytes and low density granulocytes can be distinguished and may have very different roles [49]. Whether this is true in preeclampsia is yet to be determined.

In the rat as in the human, neutrophils have been shown to increase in circulation over the course of a normal pregnancy. The primary goal of our study was to determine if the neutrophil was necessary or permissive for placental ischemia-induced hypertension. Many cell types including neutrophils, macrophages, endothelial cells, smooth muscle cells and T cells can convert molecular oxygen into reactive oxygen species that contribute to disease pathology. In our study, oxidative stress was measured because it had been previously demonstrated to be important in hypertension in this model, and if neutrophils were contributing to the oxidative stress, then neutrophil depletion would have reduced the oxidative stress. However, if oxidative stress occurred regardless of neutrophil depletion it would suggest another cell type(s) was important in leading to the oxidative stress and hypertension. Oxidative stress in the kidney and placenta was not evident in our study. However, a clear attenuation of hypertension was noted when neutrophils in circulation and tissues were depleted during placental ischemia. It is also important to note that neutrophil depletion did not alter blood pressure in the sham group, suggesting that the normal pregnancy-related increase in neutrophils does not play a role in the blood pressure changes that occur during uncomplicated pregnancies.

Markers of oxidative stress have been measured in numerous clinical and experimental studies and many studies demonstrate increased oxidative stress/decreased anti-oxidant capacity. Increased oxidative stress has been measured in numerous studies in the rat RUPP model [46, 50] including a study from our own group [41]. Sedeek et al demonstrated that the hypertensive response following placental ischemia was in part due to oxidative stress since the superoxide dismutase mimetic Tempol attenuated the hypertension [50] and measures of oxidative stress were increased with placental ischemia. There are also studies that do not support a generalized state of oxidative stress in women with preeclampsia [51] or indicate small but not significant or inconsistent changes in animal models [52, 53]. Thus the lack of changes in oxidative stress reported in our present study are not unique. A variety of factors could contribute to the difference including using a normal pregnant rat as control versus a surgical sham control. Our studies always used a sham surgery as control for comparison to animals with placental ischemia. Sham surgery might increase the baseline oxidative stress making it more difficult to detect an increase with placental ischemia. In addition, our current study and the studies of Lillegard et al [31, 32] did not use heparin to maintain patency of the cannulas, and heparin is known to influence the localization and binding of extracellular superoxide dismutase [54] and increase circulating myeloperoxidase [35] and sFlt-1 [36]. We also did not directly compare whether our control treatment with normal rabbit serum had an effect on oxidative stress in the animal compared to a normal pregnant animal with no treatment. A difference in the degree of oxidative stress in rats from different suppliers cannot be discounted since previous studies directly compared blood pressure responses and susceptibility to nephropathy in rats from the two suppliers and found differences [55–57]. Our studies have used Sprague Dawley rats from Charles River whereas most studies in the literature have used Sprague Dawley rats supplied by Harlan. We reported an increase in plasma MDA and placental catalase using Sprague Dawley rats from Charles River, but this study also differed from the current study in the use of heparin to maintain patency of cannulas [41]. These data suggest it is not the animal source that is responsible for the lack of a change in oxidative stress in the current study

Previous studies demonstrated increased myeloperoxidase in the placenta of RUPP rats at 20.5 dpc, suggesting the presence of increased numbers of neutrophils [50] as the pregnancy approached parturition. Our studies did not provide any evidence that neutrophils were increased in the placenta at 19.5 dpc using immunohistochemistry. To our knowledge, neutrophil activation has not been demonstrated in the rat RUPP model. In our study, we have controlled for other unspecified or off target effects of antiPMN administration by treating Sham animals with the same antibody and determining the effect on blood pressure. In addition, we verified the specificity of the treatment in vivo by monitoring platelets, hematocrit and other white blood cell populations in antiPMN and control treated animals. It is certainly possible that antibody coated neutrophils could contribute to complement activation, and neutrophil removal via the reticuloendothelial system could result in cytokine production. Our previous work demonstrated the importance of complement activation, particularly C3a and C5a, in placental ischemia-induced hypertension in the rat RUPP model using an inhibitor of complement activation as well as receptor antagonists [32]. In the current study, we saw no effect of the antibody treatment on complement activation assessed by C3a generation, and also verified that antiPMN treatment did not deplete complement by excessive activation. Receptors for C3a and C5a are on multiple cell types, including neutrophils, with C5a clearly being able to activate neutrophils and recent studies indicating that C3a inhibits neutrophil mobilization [58–61]. As designed, our study cannot determine if C3a and/or C5a action on neutrophils in some way leads to placental ischemia-induced hypertension.

Exactly how the neutrophil contributes to placental ischemia-induced hypertension or is permissive for placental ischemia-induced hypertension remains unclear. Neutrophils may impact endothelial dysfunction following placental ischemia and this endpoint was not assessed in this particular group of studies. Likewise, since endothelin can activate neutrophils [62] and has also been implicated in placental ischemia-induced hypertension [63], future studies to determine if there is a link between endothelin and neutrophils in placental ischemia-induced hypertension is also worthy of consideration.

Preeclampsia is associated with angiogenic imbalance, and previous studies by Gilbert et al [33, 38] have demonstrated that a plasma angiogenic imbalance is present in the RUPP model of placental ischemia-induced hypertension. Gilbert et al [33] demonstrated that infusion of recombinant VEGF in the RUPP model reversed the placental ischemia-induced hypertension and restored the plasma free VEGF as measured by the ELISA. In addition, Gilbert et al [38] reported that spike and recovery and dilution experiments were done with the available recombinant mouse VEGF (>95% homology with rat) and the mouse VEGF ELISA assay to verify that VEGF was being quantitated in the rat RUPP model. In the present study, we demonstrated a decrease in plasma free VEGF in RUPP animals compared to control, and this decrease in VEGF occurred despite a significant depletion of neutrophils. These data suggest that neutrophils are an important step in placental ischemia-induced hypertension as a result of angiogenic imbalance or, alternatively, that they operate in parallel to cause hypertension. Interrelationships of leukocytes and angiogenic balance have clearly been demonstrated in animal models of adverse pregnancy outcomes [26, 64], but whether these are operative following placental ischemia is not clear from our studies. Since completion of these studies, recent work has raised questions regarding the validity of the VEGF ELISA [42] used by us and others [31, 53, 65] to measure circulating rat VEGF. This issue is also observed when measuring plasma VEGF in sheep, another species widely used in studies of pregnancy and parturition [66]. Viewed in concert, it appears there may be species dependent differences in circulating VEGF during pregnancy [42, 66]. Alternatively, the differences observed between species regarding plasma VEGF concentration may be related to the sensitivity and specificity of the assay and reagents. Nevertheless, future studies will require the development of more robust assays for the quantitation of VEGF in animal models studying events during pregnancy. A decrease in circulating platelets is often associated with preeclampsia in the life threatening HELLP syndrome (Hemolysis Elevated Liver Enzymes Low Platelets). Platelets are also implicated in the production of VEGF. While the present study does not reveal any changes in platelets associated with antiPMN treatment, it does suggest that the reduction in VEGF in this model is not due to a decrease in platelets.

Neutrophil depletion did not affect the resorption rate of fetuses with the RUPP procedure though the blood pressure change was tempered. To our knowledge, the only studies in the rat RUPP model to report an improvement in fetal status are with low dose VEGF infusion [33] and in a recent study by Cornelius investigating the role of IL17 [52]. In contrast to our present findings, researchers using a mouse model of antibody-dependent miscarriage to study antiphospholipid syndrome [26, 67] reported that neutrophil depletion prevented fetal demise. In addition, the importance of the neutrophil in fetal demise was also demonstrated in the CBAxDBA/2 model of recurrent spontaneous abortion [25]. It is also important to note the differences in the timing of experimental treatments between the present study and that of the mouse models of pregnancy loss. In the present study we are investigating later pregnancy phenomena and the treatments/interventions occur later in gestation, while Girardi et al [26] and Clark et al [25] studied earlier pregnancy events in the mouse.

Our study is limited by the fact that the role of neutrophils in RUPP hypertension is only being addressed once placental ischemia has occurred. That is, the involvement of neutrophils in contributing to the development and progression of placental ischemia that precede preeclampsia is not addressed and is limited by the lack of appropriate animal models. In addition, total elimination of neutrophils is not feasible, and as seen in our monitoring of depletion of neutrophils in the circulation, a surge in circulating neutrophils occurs prior to necropsy at 19.5 dpc, despite the administration of antiPMN antibody on 18.5 dpc. This could be due to a normal increase in circulating neutrophils as the animal approaches parturition [68], the development of antibodies to the treatment (which may limit effectiveness), an acute response to the cannulation surgery on 18.5 dpc, or some combination. Studies in a limited number of animals indicate that neutrophils did not substantially increase on 19.5 dpc if carotid cannulation did not occur. Also, neutrophils did not increase on 19.5 dpc in animals that only underwent a sham surgical procedure with no placement of the carotid cannula. Thus, a significant acute effect of the surgical intervention on immune and inflammatory factors must be considered in studies like this. Regardless, the number of neutrophils in the placenta and lung were decreased with antiPMN treatment.

The effect of neutrophil depletion on elevated blood pressure is statistically significant which does not necessarily speak to biological relevance of the magnitude of the effect. This attenuation of blood pressure rather than complete inhibition is not unique to neutrophil depletion in the RUPP model. For example, inhibition of IL17 [52], B cells and CD4 T cells [44, 46], TNF-α [69, 70], using pravastatin [41] and inhibition of complement activation and antagonizing C3a and C5a receptors [31, 32] all *partially* inhibit the RUPP induced increase in blood pressure. Our data and previous studies suggest that a variety of innate and adaptive immune cells and mediators play a role in placental ischemia-induced hypertension. The endothelin receptor antagonist atrasentan and the angiotensin type I receptor inhibitor losartan lower blood pressure to normal pregnant levels in this rat model of placental ischemia induced hypertension, suggesting that they affect multiple steps that contribute to hypertension [63, 71].

Overall these studies are the first to implicate the neutrophil as important in placental ischemia-induced hypertension in the RUPP rat model. This coupled with the literature demonstrating the importance of lymphocytes in pregnancy-induced hypertension indicates that both innate and adaptive immunity are important effectors. This is consistent with the importance of controlling both the innate and adaptive immune system to insure a successful pregnancy. Thus, therapeutic intervention in pregnancy-induced hypertension may require a broad approach targeting both innate and adaptive immunity.

## Supporting Information

S1 Fig Treatment with antiPMN antibody on 13.5–18.5 dpc does not affect numbers of circulating platelets. Animals were treated with normal rabbit serum (Control) or antiPMN antibody as in Fig 5. AntiPMN treatment did not alter platelet numbers over the time course of the experiment in antiPMN compared to control. Values represent mean ± SE of platelets/ml in blood collected from 5–14 animals as described in Methods.
(TIF)

S2 Fig Treatment with antiPMN antibody on 13.5–18.5 dpc does not affect the number of circulating lymphocytes. Animals were treated with normal rabbit serum (Control) or antiPMN antibody as in Fig 5. AntiPMN treatment resulted in an increase in the % lymphocytes due to neutrophil depletion. Values represent mean ± SE of % lymphocytes in blood collected from 5–14 animals as described in Methods.
(TIF)

S1 File Spreadsheet of data used in figs and text. (XLSX)

S2 File Data summary of circulating cell counts used in determining effectiveness of antiPMN treatment. (XLSX)
