# Supplementary figures and images for: Neutrophil Depletion Attenuates Placental Ischemia-Induced Hypertension in the Rat
Source: PLoS One. 2015 Jul 2;10(7):e0132063. doi: 10.1371/journal.pone.0132063 (PMC4509576; doi:10.1371/journal.pone.0132063)

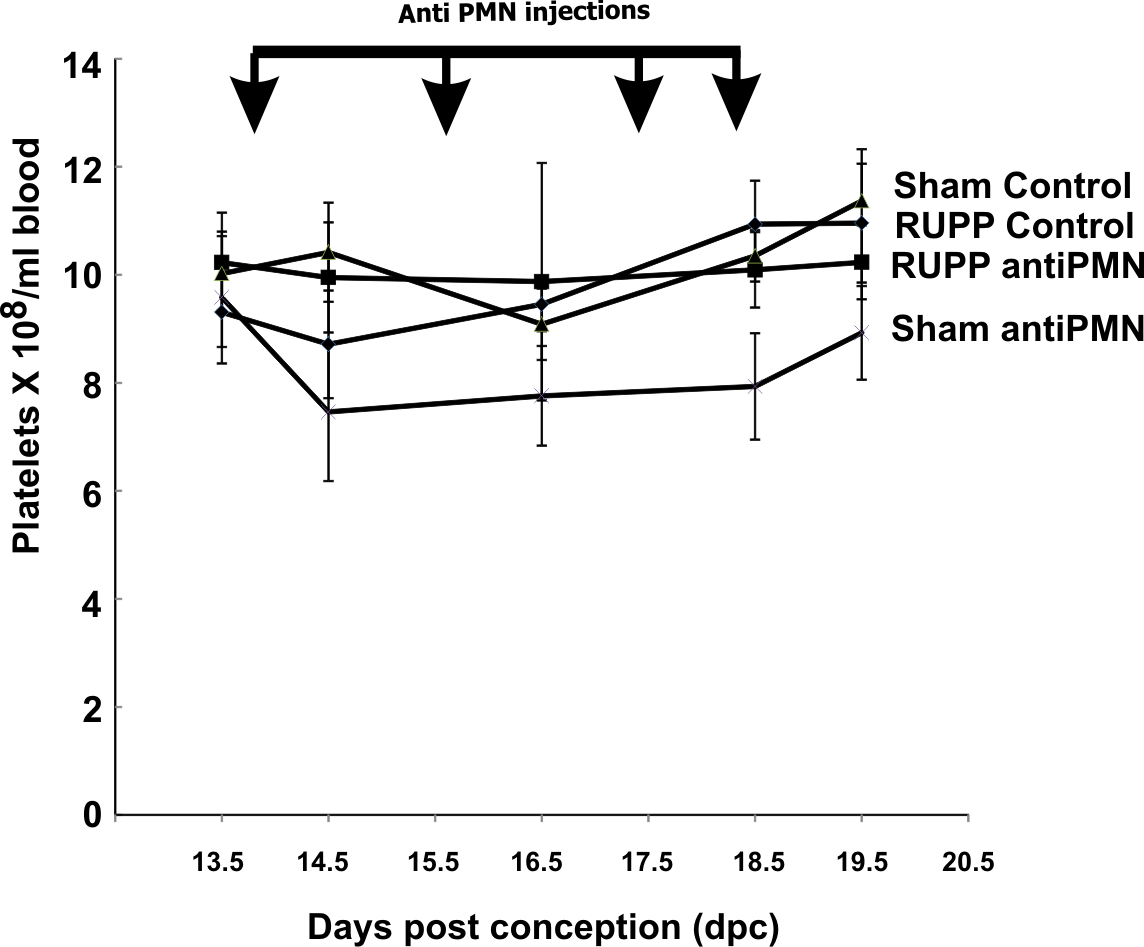

Supplement: S1 Fig — Animals were treated with normal rabbit serum (Control) or antiPMN antibody as in Fig 5. AntiPMN treatment did not alter platelet numbers over the time course of the experiment in antiPMN compared to control. Values represent mean ± SE of platelets/ml in blood collected from 5–14 animals as described in Methods. (TIF) [file pone.0132063.s001.tif]

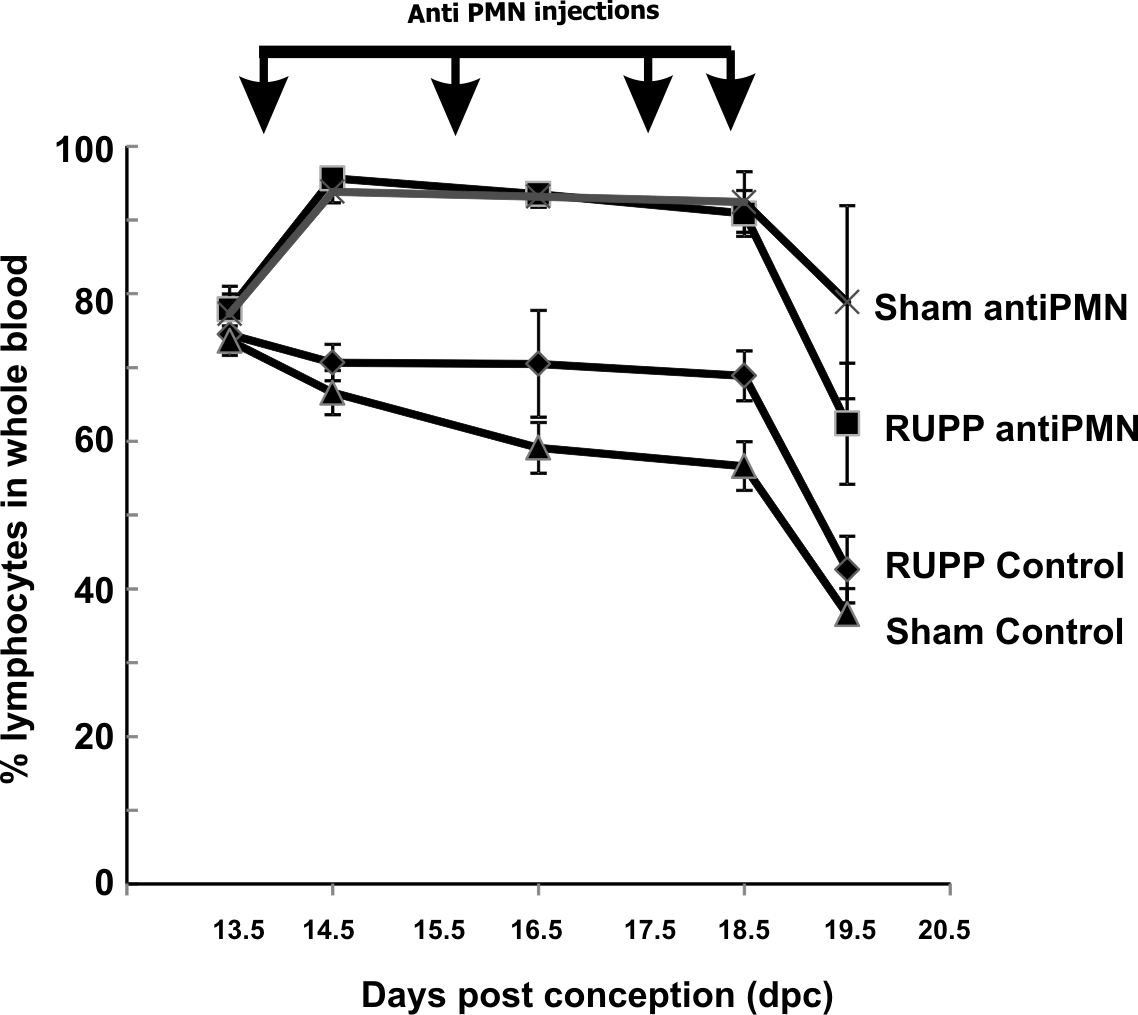

Supplement: S2 Fig — Animals were treated with normal rabbit serum (Control) or antiPMN antibody as in Fig 5. AntiPMN treatment resulted in an increase in the % lymphocytes due to neutrophil depletion. Values represent mean ± SE of % lymphocytes in blood collected from 5–14 animals as described in Methods. (TIF) [file pone.0132063.s002.tif]
